# Supplementary material for: The influence of gender stereotypes on gender judgement and impression evaluation based on face and voice
Source: PeerJ. 2025 Jan 31;13:e18900. doi: 10.7717/peerj.18900 (PMC11789659; doi:10.7717/peerj.18900)
Supplement: Supplemental Information 5 [file peerj-13-18900-s005.docx]

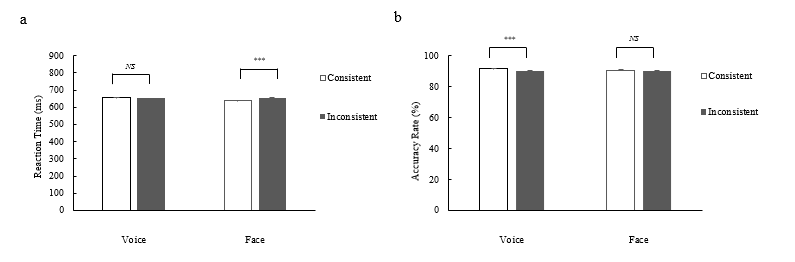


*Note*：^***^：*p* < 0 .001; ^**^：*p* < 0 .01; *NS*：*p* > 0.05

Figure 2 The effect of perceptual targets on reaction time (a) and accuracy (b) under consistent and inconsistent conditions


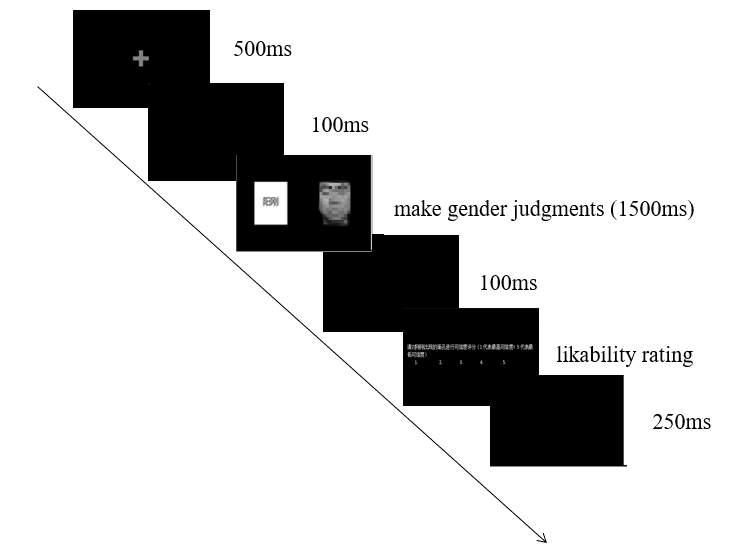


*Note*：The face in this image has been blurred for publication purposes. In the original experiment, recognizable faces were used.

Figure 1 A flowchart of an experimental trial. Face stimuli were obtained from the Chinese Affective Face Picture System (Gong et al., 2011), for which the Department of Psychology at Tianjin Normal University has purchased the copyright; Sound stimuli were sourced from the open-source Chinese Affective Sound Inventory (available at <https://gitcode.com/open-source> -toolkit/115f2/overview?utm_source=tools_gitcode&index=top&type=card& ).
