# Supplementary material for: The influence of gender stereotypes on gender judgement and impression evaluation based on face and voice
Source: PeerJ. 2025 Jan 31;13:e18900. doi: 10.7717/peerj.18900 (PMC11789659; doi:10.7717/peerj.18900)
Supplement: Supplemental Information 7 [file peerj-13-18900-s007.docx]

**Experimental Results based on stimulus-level analysis**

The results of the subject-level analyses are reported in the official manuscript, and in the Supplementary Material we further provide the results of the stimulus-level analyses. The independent and dependent variables for this part of the analysis methods are consistent with those in the official manuscript. Specific results are presented below:

Statistical tests using reaction time as the dependent variable revealed a significant main effect of perceptual targets, the reaction time for gender judgements based on faces is shorter than that for voice judgements, *F*(1, 87) = 92.57, *p* < 0.001, partial η^2^ = 0.03. The main effect of gender stereotype information was also significant, the reaction time is shorter when gender stereotype information is consistent than when they are inconsistent, *F*(1, 87) = 25.54, *p* < 0.001, partial η^2^ = 0.01. The main effect of the gender of the perceptual target is not significant. The interaction between perceptual targets and gender stereotype information was particularly significant, *F*(1, 87) =82.6, *p* < 0.001, partial η^2^ = 0.03. A simple effects analysis indicated that when the perceptual target was voices, there was no significant difference in reaction time between the consistent and inconsistent conditions; however, when the perceptual target was faces, reaction time increased in the inconsistent condition compared to the consistent condition, *t*(174) = 9.77, *p* < 0.001, Cohen’*d* = 0.19*.*

The statistical examination, with accuracy rate as the key dependent measure, did not yield a significant main effect for the perceptual target. In contrast, the main effect for gender stereotype information was significant, the accuracy rate is higher when gender stereotype information is consistent than when they are inconsistent, *F*(1, 87) = 5.09, *p* < 0.05, partial η^2^ = 0.02. The main effect of the gender of the perceptual target is not significant. The interaction effect between the perceptual target and gender stereotype information was found to be significant, *F*(1,87) = 3.92, *p* < 0.05, partial η^2^ = 0.001. A simple effects analysis showed that when the perceptual target was voices, accuracy increased significantly in the consistent conditions compared to the inconsistent conditions, *t*(174) = 2.98，*p* < 0.003，Cohen’*d* = 0.07，whereas when the perceptual target was faces, there was no significant difference in accuracy between consistent and inconsistent conditions.

The statistical examination, which employed likability ratings as the dependent measure, did not detect a significant main effect for the perceptual target and the perceptual target’s gender. In contrast, the influence of gender stereotype information was found to be statistically significant, the likability ratings is higher when gender stereotype information is consistent than when they are inconsistent, *F*(1, 87) = 31.25, *p* < 0.001, partial η^2^ = 0.012. The interaction effect between the perceptual target and gender stereotype information did not reach statistical significance.
