# Supplementary material for: The influence of gender stereotypes on gender judgement and impression evaluation based on face and voice
Source: PeerJ. 2025 Jan 31;13:e18900. doi: 10.7717/peerj.18900 (PMC11789659; doi:10.7717/peerj.18900)
Supplement: Supplemental Information 8 [file peerj-13-18900-s008.docx]

**Gender Trait Word Open Survey Questionnaire**

Dear Student,

Hello! This is a questionnaire about people's impressions of males and females. Please take a moment out of your busy schedule to complete this survey. Thank you!

| Sex |  | | | Age | |  | |
| --- | --- | --- | --- | --- | --- | --- | --- |
| Major |  | | | Grade | |  | |
| (1) Please list some two-character words that are often used to describe typical male characteristics (they can be adjectives, nouns, or verbs; at least 15 words, the more the better). | | | | | | | |
| ___________ | | ___________ | ___________ | | ___________ | | ___________ |
| ___________ | | ___________ | ___________ | | ___________ | | ___________ |
| ___________ | | ___________ | ___________ | | ___________ | | ___________ |
| ___________ | | ___________ | ___________ | | ___________ | | ___________ |
| ___________ | | ___________ | ___________ | | ___________ | | ___________ |
| ___________ | | ___________ | ___________ | | ___________ | | ___________ |
| (2) Please list some two-character words that are often used to describe typical female characteristics (they can be adjectives, nouns, or verbs; at least 15 words, the more the better). | | | | | | | |
| ___________ | | ___________ | ___________ | | ___________ | | ___________ |
| ___________ | | ___________ | ___________ | | ___________ | | ___________ |
| ___________ | | ___________ | ___________ | | ___________ | | ___________ |
| ___________ | | ___________ | ___________ | | ___________ | | ___________ |
| ___________ | | ___________ | ___________ | | ___________ | | ___________ |
| ___________ | | ___________ | ___________ | | ___________ | | ___________ |

Thank you very much for your assistance! I wish you progress in your studies and a pleasant life!
