# Supplementary material for: The influence of gender stereotypes on gender judgement and impression evaluation based on face and voice
Source: PeerJ. 2025 Jan 31;13:e18900. doi: 10.7717/peerj.18900 (PMC11789659; doi:10.7717/peerj.18900)
Supplement: Supplemental Information 9 [file peerj-13-18900-s009.docx]

**性别特质词开放式调查问卷**

亲爱的同学：您好！这是一份关于人们对男性、女性印象的问卷，请您在百忙之中抽出一点时间完成本次调查。谢谢！

| 性别 |  | | | 年龄 | |  | |
| --- | --- | --- | --- | --- | --- | --- | --- |
| 专业 |  | | | 年级 | |  | |
| (1)请您根据印象写出一些经常用来描述男性典型特征的双字词(可以是 形容词、名词或动词；至少 15 个，越多越好)。 | | | | | | | |
| ___________ | | ___________ | ___________ | | ___________ | | ___________ |
| ___________ | | ___________ | ___________ | | ___________ | | ___________ |
| ___________ | | ___________ | ___________ | | ___________ | | ___________ |
| ___________ | | ___________ | ___________ | | ___________ | | ___________ |
| ___________ | | ___________ | ___________ | | ___________ | | ___________ |
| ___________ | | ___________ | ___________ | | ___________ | | ___________ |
| (2)请您根据印象写出一些经常用来描述女性典型特征的双字词(可以是 形容词、名词或动词；至少 15 个，越多越好)。 | | | | | | | |
| ___________ | | ___________ | ___________ | | ___________ | | ___________ |
| ___________ | | ___________ | ___________ | | ___________ | | ___________ |
| ___________ | | ___________ | ___________ | | ___________ | | ___________ |
| ___________ | | ___________ | ___________ | | ___________ | | ___________ |
| ___________ | | ___________ | ___________ | | ___________ | | ___________ |
| ___________ | | ___________ | ___________ | | ___________ | | ___________ |

非常感谢您的帮助！祝您学习进步，生活愉快！
