# Supplementary material for: The influence of gender stereotypes on gender judgement and impression evaluation based on face and voice
Source: PeerJ. 2025 Jan 31;13:e18900. doi: 10.7717/peerj.18900 (PMC11789659; doi:10.7717/peerj.18900)
Supplement: Supplemental Information 10 [file peerj-13-18900-s010.docx]

**性别特质词典型性问卷**

性别___________ 年龄___________ 年级___________ 专业_____________

亲爱的同学： 下面是一些经常用来描述男性或女性的词语，请您根据它们适合描述男性或适合描述女性的 程度进行评分。其中，1 代表男性化程度最强，即“非常男性化”；2 代表“比较男性化”；依此类推， 3 代表的男性化程度更低；4 代表“中性化”，即这一特征对于男女均可；5 代表偏向女性化；6 代 表“比较女性化”；7 代表女性化程度最强，即“非常女性化”。答案无对错之分，仅用于研究。请 您根据自己的真实看法认真评价，并在相应得分上打 “√”。

| 序 号 非常男性化 中性化 非常女性化 | | | | | | | | |
| --- | --- | --- | --- | --- | --- | --- | --- | --- |
| 1 | 高大 | 1 | 2 | 3 | 4 | 5 | 6 | 7 |
| 2 | 爱心 | 1 | 2 | 3 | 4 | 5 | 6 | 7 |
| 3 | 安静 | 1 | 2 | 3 | 4 | 5 | 6 | 7 |
| 4 | 保守 | 1 | 2 | 3 | 4 | 5 | 6 | 7 |
| 5 | 抱负 | 1 | 2 | 3 | 4 | 5 | 6 | 7 |
| 6 | 暴躁 | 1 | 2 | 3 | 4 | 5 | 6 | 7 |
| 7 | 被动 | 1 | 2 | 3 | 4 | 5 | 6 | 7 |
| 8 | 沉默 | 1 | 2 | 3 | 4 | 5 | 6 | 7 |
| 9 | 冲动 | 1 | 2 | 3 | 4 | 5 | 6 | 7 |
| 10 | 粗鲁 | 1 | 2 | 3 | 4 | 5 | 6 | 7 |
| 11 | 大方 | 1 | 2 | 3 | 4 | 5 | 6 | 7 |
| 12 | 低微 | 1 | 2 | 3 | 4 | 5 | 6 | 7 |
| 13 | 独立 | 1 | 2 | 3 | 4 | 5 | 6 | 7 |
| 14 | 感性 | 1 | 2 | 3 | 4 | 5 | 6 | 7 |
| 15 | 刚强 | 1 | 2 | 3 | 4 | 5 | 6 | 7 |
| 16 | 刚毅 | 1 | 2 | 3 | 4 | 5 | 6 | 7 |
| 17 | 刚正 | 1 | 2 | 3 | 4 | 5 | 6 | 7 |
| 18 | 公益 | 1 | 2 | 3 | 4 | 5 | 6 | 7 |
| 19 | 攻击 | 1 | 2 | 3 | 4 | 5 | 6 | 7 |
| 20 | 关怀 | 1 | 2 | 3 | 4 | 5 | 6 | 7 |
| 21 | 真诚 | 1 | 2 | 3 | 4 | 5 | 6 | 7 |
| 22 | 果断 | 1 | 2 | 3 | 4 | 5 | 6 | 7 |
| 23 | 霸道 | 1 | 2 | 3 | 4 | 5 | 6 | 7 |
| 24 | 霸气 | 1 | 2 | 3 | 4 | 5 | 6 | 7 |
| 25 | 沉稳 | 1 | 2 | 3 | 4 | 5 | 6 | 7 |
| 26 | 成熟 | 1 | 2 | 3 | 4 | 5 | 6 | 7 |
| 27 | 高尚 | 1 | 2 | 3 | 4 | 5 | 6 | 7 |
| 28 | 果敢 | 1 | 2 | 3 | 4 | 5 | 6 | 7 |
| 29 | 贤淑 | 1 | 2 | 3 | 4 | 5 | 6 | 7 |
| 30 | 孝顺 | 1 | 2 | 3 | 4 | 5 | 6 | 7 |
| 31 | 羞花 | 1 | 2 | 3 | 4 | 5 | 6 | 7 |
| 32 | 羞涩 | 1 | 2 | 3 | 4 | 5 | 6 | 7 |
| 33 | 秀丽 | 1 | 2 | 3 | 4 | 5 | 6 | 7 |
| 34 | 秀气 | 1 | 2 | 3 | 4 | 5 | 6 | 7 |
| 35 | 秀雅 | 1 | 2 | 3 | 4 | 5 | 6 | 7 |
| 36 | 窈窕 | 1 | 2 | 3 | 4 | 5 | 6 | 7 |
| 37 | 依附 | 1 | 2 | 3 | 4 | 5 | 6 | 7 |
| 38 | 依赖 | 1 | 2 | 3 | 4 | 5 | 6 | 7 |
| 39 | 阴柔 | 1 | 2 | 3 | 4 | 5 | 6 | 7 |
| 40 | 优雅 | 1 | 2 | 3 | 4 | 5 | 6 | 7 |
| 41 | 好斗 | 1 | 2 | 3 | 4 | 5 | 6 | 7 |
| 42 | 野蛮 | 1 | 2 | 3 | 4 | 5 | 6 | 7 |
| 43 | 冒险 | 1 | 2 | 3 | 4 | 5 | 6 | 7 |
| 44 | 敌意 | 1 | 2 | 3 | 4 | 5 | 6 | 7 |
| 45 | 鲁莽 | 1 | 2 | 3 | 4 | 5 | 6 | 7 |
| 46 | 挑战 | 1 | 2 | 3 | 4 | 5 | 6 | 7 |
| 47 | 凶狠 | 1 | 2 | 3 | 4 | 5 | 6 | 7 |
| 48 | 狂怒 | 1 | 2 | 3 | 4 | 5 | 6 | 7 |
| 49 | 谨慎 | 1 | 2 | 3 | 4 | 5 | 6 | 7 |
| 50 | 忍耐 | 1 | 2 | 3 | 4 | 5 | 6 | 7 |
| 51 | 逃避 | 1 | 2 | 3 | 4 | 5 | 6 | 7 |
| 52 | 亲和 | 1 | 2 | 3 | 4 | 5 | 6 | 7 |
| 53 | 宽容 | 1 | 2 | 3 | 4 | 5 | 6 | 7 |
| 54 | 诚实 | 1 | 2 | 3 | 4 | 5 | 6 | 7 |
| 55 | 友好 | 1 | 2 | 3 | 4 | 5 | 6 | 7 |
| 56 | 幽默 | 1 | 2 | 3 | 4 | 5 | 6 | 7 |
| 57 | 憨厚 | 1 | 2 | 3 | 4 | 5 | 6 | 7 |
| 58 | 豪放 | 1 | 2 | 3 | 4 | 5 | 6 | 7 |
| 59 | 豪气 | 1 | 2 | 3 | 4 | 5 | 6 | 7 |
| 60 | 豪言 | 1 | 2 | 3 | 4 | 5 | 6 | 7 |
| 61 | 积极 | 1 | 2 | 3 | 4 | 5 | 6 | 7 |
| 62 | 激进 | 1 | 2 | 3 | 4 | 5 | 6 | 7 |
| 63 | 急躁 | 1 | 2 | 3 | 4 | 5 | 6 | 7 |
| 64 | 坚定 | 1 | 2 | 3 | 4 | 5 | 6 | 7 |
| 65 | 坚强 | 1 | 2 | 3 | 4 | 5 | 6 | 7 |
| 66 | 坚毅 | 1 | 2 | 3 | 4 | 5 | 6 | 7 |
| 67 | 健壮 | 1 | 2 | 3 | 4 | 5 | 6 | 7 |
| 68 | 进取 | 1 | 2 | 3 | 4 | 5 | 6 | 7 |
| 69 | 竞争 | 1 | 2 | 3 | 4 | 5 | 6 | 7 |
| 70 | 开放 | 1 | 2 | 3 | 4 | 5 | 6 | 7 |
| 71 | 开阔 | 1 | 2 | 3 | 4 | 5 | 6 | 7 |
| 72 | 客观 | 1 | 2 | 3 | 4 | 5 | 6 | 7 |
| 73 | 魁梧 | 1 | 2 | 3 | 4 | 5 | 6 | 7 |
| 74 | 冷静 | 1 | 2 | 3 | 4 | 5 | 6 | 7 |
| 75 | 理性 | 1 | 2 | 3 | 4 | 5 | 6 | 7 |
| 76 | 谦虚 | 1 | 2 | 3 | 4 | 5 | 6 | 7 |
| 77 | 强大 | 1 | 2 | 3 | 4 | 5 | 6 | 7 |
| 78 | 强健 | 1 | 2 | 3 | 4 | 5 | 6 | 7 |
| 79 | 强势 | 1 | 2 | 3 | 4 | 5 | 6 | 7 |
| 80 | 强壮 | 1 | 2 | 3 | 4 | 5 | 6 | 7 |
| 81 | 权势 | 1 | 2 | 3 | 4 | 5 | 6 | 7 |
| 82 | 权威 | 1 | 2 | 3 | 4 | 5 | 6 | 7 |
| 83 | 儒雅 | 1 | 2 | 3 | 4 | 5 | 6 | 7 |
| 84 | 洒脱 | 1 | 2 | 3 | 4 | 5 | 6 | 7 |
| 85 | 少言 | 1 | 2 | 3 | 4 | 5 | 6 | 7 |
| 86 | 深沉 | 1 | 2 | 3 | 4 | 5 | 6 | 7 |
| 87 | 帅气 | 1 | 2 | 3 | 4 | 5 | 6 | 7 |
| 88 | 顽强 | 1 | 2 | 3 | 4 | 5 | 6 | 7 |
| 89 | 威猛 | 1 | 2 | 3 | 4 | 5 | 6 | 7 |
| 90 | 伟大 | 1 | 2 | 3 | 4 | 5 | 6 | 7 |
| 91 | 稳重 | 1 | 2 | 3 | 4 | 5 | 6 | 7 |
| 92 | 逍遥 | 1 | 2 | 3 | 4 | 5 | 6 | 7 |
| 93 | 潇洒 | 1 | 2 | 3 | 4 | 5 | 6 | 7 |
| 94 | 凶猛 | 1 | 2 | 3 | 4 | 5 | 6 | 7 |
| 95 | 严肃 | 1 | 2 | 3 | 4 | 5 | 6 | 7 |
| 96 | 阳刚 | 1 | 2 | 3 | 4 | 5 | 6 | 7 |
| 97 | 阳光 | 1 | 2 | 3 | 4 | 5 | 6 | 7 |
| 98 | 英俊 | 1 | 2 | 3 | 4 | 5 | 6 | 7 |
| 99 | 英勇 | 1 | 2 | 3 | 4 | 5 | 6 | 7 |
| 100 | 硬气 | 1 | 2 | 3 | 4 | 5 | 6 | 7 |
| 101 | 勇敢 | 1 | 2 | 3 | 4 | 5 | 6 | 7 |
| 102 | 强硬 | 1 | 2 | 3 | 4 | 5 | 6 | 7 |
| 103 | 主动 | 1 | 2 | 3 | 4 | 5 | 6 | 7 |
| 104 | 主见 | 1 | 2 | 3 | 4 | 5 | 6 | 7 |
| 105 | 快乐 | 1 | 2 | 3 | 4 | 5 | 6 | 7 |
| 106 | 博学 | 1 | 2 | 3 | 4 | 5 | 6 | 7 |
| 107 | 理智 | 1 | 2 | 3 | 4 | 5 | 6 | 7 |
| 108 | 真挚 | 1 | 2 | 3 | 4 | 5 | 6 | 7 |
| 109 | 自重 | 1 | 2 | 3 | 4 | 5 | 6 | 7 |
| 110 | 多才 | 1 | 2 | 3 | 4 | 5 | 6 | 7 |
| 111 | 坦诚 | 1 | 2 | 3 | 4 | 5 | 6 | 7 |
| 112 | 好学 | 1 | 2 | 3 | 4 | 5 | 6 | 7 |
| 113 | 勤劳 | 1 | 2 | 3 | 4 | 5 | 6 | 7 |
| 114 | 礼貌 | 1 | 2 | 3 | 4 | 5 | 6 | 7 |
| 115 | 慷慨 | 1 | 2 | 3 | 4 | 5 | 6 | 7 |
| 116 | 认真 | 1 | 2 | 3 | 4 | 5 | 6 | 7 |
| 117 | 热情 | 1 | 2 | 3 | 4 | 5 | 6 | 7 |
| 118 | 刻苦 | 1 | 2 | 3 | 4 | 5 | 6 | 7 |
| 119 | 乐观 | 1 | 2 | 3 | 4 | 5 | 6 | 7 |
| 120 | 敬业 | 1 | 2 | 3 | 4 | 5 | 6 | 7 |
| 121 | 谦逊 | 1 | 2 | 3 | 4 | 5 | 6 | 7 |
| 122 | 仁慈 | 1 | 2 | 3 | 4 | 5 | 6 | 7 |
| 123 | 自尊 | 1 | 2 | 3 | 4 | 5 | 6 | 7 |
| 124 | 自爱 | 1 | 2 | 3 | 4 | 5 | 6 | 7 |
| 125 | 自主 | 1 | 2 | 3 | 4 | 5 | 6 | 7 |
| 126 | 稳进 | 1 | 2 | 3 | 4 | 5 | 6 | 7 |
| 127 | 安详 | 1 | 2 | 3 | 4 | 5 | 6 | 7 |
| 128 | 慈善 | 1 | 2 | 3 | 4 | 5 | 6 | 7 |
| 129 | 美好 | 1 | 2 | 3 | 4 | 5 | 6 | 7 |
| 130 | 吉祥 | 1 | 2 | 3 | 4 | 5 | 6 | 7 |
| 131 | 开朗 | 1 | 2 | 3 | 4 | 5 | 6 | 7 |
| 132 | 恶毒 | 1 | 2 | 3 | 4 | 5 | 6 | 7 |
| 133 | 阿谀 | 1 | 2 | 3 | 4 | 5 | 6 | 7 |
| 134 | 卑鄙 | 1 | 2 | 3 | 4 | 5 | 6 | 7 |
| 135 | 卑劣 | 1 | 2 | 3 | 4 | 5 | 6 | 7 |
| 136 | 悲伤 | 1 | 2 | 3 | 4 | 5 | 6 | 7 |
| 137 | 愤怒 | 1 | 2 | 3 | 4 | 5 | 6 | 7 |
| 138 | 讽刺 | 1 | 2 | 3 | 4 | 5 | 6 | 7 |
| 139 | 负义 | 1 | 2 | 3 | 4 | 5 | 6 | 7 |
| 140 | 孤立 | 1 | 2 | 3 | 4 | 5 | 6 | 7 |
| 141 | 狠毒 | 1 | 2 | 3 | 4 | 5 | 6 | 7 |
| 142 | 昏庸 | 1 | 2 | 3 | 4 | 5 | 6 | 7 |
| 143 | 奸诈 | 1 | 2 | 3 | 4 | 5 | 6 | 7 |
| 144 | 刻薄 | 1 | 2 | 3 | 4 | 5 | 6 | 7 |
| 145 | 毛躁 | 1 | 2 | 3 | 4 | 5 | 6 | 7 |
| 146 | 难过 | 1 | 2 | 3 | 4 | 5 | 6 | 7 |
| 147 | 奴性 | 1 | 2 | 3 | 4 | 5 | 6 | 7 |
| 148 | 欺人 | 1 | 2 | 3 | 4 | 5 | 6 | 7 |
| 149 | 失败 | 1 | 2 | 3 | 4 | 5 | 6 | 7 |
| 150 | 失落 | 1 | 2 | 3 | 4 | 5 | 6 | 7 |
| 151 | 失望 | 1 | 2 | 3 | 4 | 5 | 6 | 7 |
| 152 | 痛苦 | 1 | 2 | 3 | 4 | 5 | 6 | 7 |
| 153 | 自负 | 1 | 2 | 3 | 4 | 5 | 6 | 7 |
| 154 | 自信 | 1 | 2 | 3 | 4 | 5 | 6 | 7 |
| 155 | 百媚 | 1 | 2 | 3 | 4 | 5 | 6 | 7 |
| 156 | 闭月 | 1 | 2 | 3 | 4 | 5 | 6 | 7 |
| 157 | 纯真 | 1 | 2 | 3 | 4 | 5 | 6 | 7 |
| 158 | 典雅 | 1 | 2 | 3 | 4 | 5 | 6 | 7 |
| 159 | 端庄 | 1 | 2 | 3 | 4 | 5 | 6 | 7 |
| 160 | 丰润 | 1 | 2 | 3 | 4 | 5 | 6 | 7 |
| 161 | 高贵 | 1 | 2 | 3 | 4 | 5 | 6 | 7 |
| 162 | 害羞 | 1 | 2 | 3 | 4 | 5 | 6 | 7 |
| 163 | 含羞 | 1 | 2 | 3 | 4 | 5 | 6 | 7 |
| 164 | 和气 | 1 | 2 | 3 | 4 | 5 | 6 | 7 |
| 165 | 佳人 | 1 | 2 | 3 | 4 | 5 | 6 | 7 |
| 166 | 娇小 | 1 | 2 | 3 | 4 | 5 | 6 | 7 |
| 167 | 矜持 | 1 | 2 | 3 | 4 | 5 | 6 | 7 |
| 168 | 可爱 | 1 | 2 | 3 | 4 | 5 | 6 | 7 |
| 169 | 绝色 | 1 | 2 | 3 | 4 | 5 | 6 | 7 |
| 170 | 克制 | 1 | 2 | 3 | 4 | 5 | 6 | 7 |
| 171 | 灵巧 | 1 | 2 | 3 | 4 | 5 | 6 | 7 |
| 172 | 美丽 | 1 | 2 | 3 | 4 | 5 | 6 | 7 |
| 173 | 腼腆 | 1 | 2 | 3 | 4 | 5 | 6 | 7 |
| 174 | 耐心 | 1 | 2 | 3 | 4 | 5 | 6 | 7 |
| 175 | 漂亮 | 1 | 2 | 3 | 4 | 5 | 6 | 7 |
| 176 | 娉婷 | 1 | 2 | 3 | 4 | 5 | 6 | 7 |
| 177 | 泼辣 | 1 | 2 | 3 | 4 | 5 | 6 | 7 |
| 178 | 内敛 | 1 | 2 | 3 | 4 | 5 | 6 | 7 |
| 179 | 千娇 | 1 | 2 | 3 | 4 | 5 | 6 | 7 |
| 180 | 俏丽 | 1 | 2 | 3 | 4 | 5 | 6 | 7 |
| 181 | 轻盈 | 1 | 2 | 3 | 4 | 5 | 6 | 7 |
| 182 | 柔美 | 1 | 2 | 3 | 4 | 5 | 6 | 7 |
| 183 | 俏皮 | 1 | 2 | 3 | 4 | 5 | 6 | 7 |
| 184 | 柔和 | 1 | 2 | 3 | 4 | 5 | 6 | 7 |
| 185 | 柔弱 | 1 | 2 | 3 | 4 | 5 | 6 | 7 |
| 186 | 善良 | 1 | 2 | 3 | 4 | 5 | 6 | 7 |
| 187 | 顺从 | 1 | 2 | 3 | 4 | 5 | 6 | 7 |
| 188 | 顺服 | 1 | 2 | 3 | 4 | 5 | 6 | 7 |
| 189 | 随和 | 1 | 2 | 3 | 4 | 5 | 6 | 7 |
| 190 | 体贴 | 1 | 2 | 3 | 4 | 5 | 6 | 7 |
| 191 | 婉顺 | 1 | 2 | 3 | 4 | 5 | 6 | 7 |
| 192 | 温柔 | 1 | 2 | 3 | 4 | 5 | 6 | 7 |
| 193 | 温顺 | 1 | 2 | 3 | 4 | 5 | 6 | 7 |
| 194 | 温婉 | 1 | 2 | 3 | 4 | 5 | 6 | 7 |
| 195 | 文静 | 1 | 2 | 3 | 4 | 5 | 6 | 7 |
| 196 | 文雅 | 1 | 2 | 3 | 4 | 5 | 6 | 7 |
| 197 | 细腻 | 1 | 2 | 3 | 4 | 5 | 6 | 7 |
| 198 | 细心 | 1 | 2 | 3 | 4 | 5 | 6 | 7 |
| 199 | 妩媚 | 1 | 2 | 3 | 4 | 5 | 6 | 7 |
| 200 | 贤惠 | 1 | 2 | 3 | 4 | 5 | 6 | 7 |
| 201 | 畏缩 | 1 | 2 | 3 | 4 | 5 | 6 | 7 |
| 202 | 污秽 | 1 | 2 | 3 | 4 | 5 | 6 | 7 |
| 203 | 无耻 | 1 | 2 | 3 | 4 | 5 | 6 | 7 |
| 204 | 狭隘 | 1 | 2 | 3 | 4 | 5 | 6 | 7 |
| 205 | 下贱 | 1 | 2 | 3 | 4 | 5 | 6 | 7 |
| 206 | 下流 | 1 | 2 | 3 | 4 | 5 | 6 | 7 |
| 207 | 凶残 | 1 | 2 | 3 | 4 | 5 | 6 | 7 |
| 208 | 消极 | 1 | 2 | 3 | 4 | 5 | 6 | 7 |
| 209 | 虚伪 | 1 | 2 | 3 | 4 | 5 | 6 | 7 |
| 210 | 阴险 | 1 | 2 | 3 | 4 | 5 | 6 | 7 |
| 211 | 愚昧 | 1 | 2 | 3 | 4 | 5 | 6 | 7 |
| 212 | 自大 | 1 | 2 | 3 | 4 | 5 | 6 | 7 |
| 213 | 自满 | 1 | 2 | 3 | 4 | 5 | 6 | 7 |
| 214 | 自私 | 1 | 2 | 3 | 4 | 5 | 6 | 7 |
| 215 | 愚蠢 | 1 | 2 | 3 | 4 | 5 | 6 | 7 |
| 216 | 自利 | 1 | 2 | 3 | 4 | 5 | 6 | 7 |
| 217 | 還遢 | 1 | 2 | 3 | 4 | 5 | 6 | 7 |
| 218 | 龌龊 | 1 | 2 | 3 | 4 | 5 | 6 | 7 |
| 219 | 糟糕 | 1 | 2 | 3 | 4 | 5 | 6 | 7 |
| 220 | 讥讽 | 1 | 2 | 3 | 4 | 5 | 6 | 7 |
| 221 | 嘲笑 | 1 | 2 | 3 | 4 | 5 | 6 | 7 |
| 222 | 轻视 | 1 | 2 | 3 | 4 | 5 | 6 | 7 |
| 223 | 蔑视 | 1 | 2 | 3 | 4 | 5 | 6 | 7 |
| 224 | 尖酸 | 1 | 2 | 3 | 4 | 5 | 6 | 7 |
| 225 | 恶意 | 1 | 2 | 3 | 4 | 5 | 6 | 7 |
| 226 | 绝望 | 1 | 2 | 3 | 4 | 5 | 6 | 7 |
| 227 | 负责 | 1 | 2 | 3 | 4 | 5 | 6 | 7 |
| 228 | 严谨 | 1 | 2 | 3 | 4 | 5 | 6 | 7 |
| 229 | 圣洁 | 1 | 2 | 3 | 4 | 5 | 6 | 7 |
| 230 | 无私 | 1 | 2 | 3 | 4 | 5 | 6 | 7 |
| 231 | 仁心 | 1 | 2 | 3 | 4 | 5 | 6 | 7 |
| 232 | 勤奋 | 1 | 2 | 3 | 4 | 5 | 6 | 7 |
| 233 | 能干 | 1 | 2 | 3 | 4 | 5 | 6 | 7 |
| 234 | 大度 | 1 | 2 | 3 | 4 | 5 | 6 | 7 |
| 235 | 宽厚 | 1 | 2 | 3 | 4 | 5 | 6 | 7 |
| 236 | 温和 | 1 | 2 | 3 | 4 | 5 | 6 | 7 |
| 237 | 德高 | 1 | 2 | 3 | 4 | 5 | 6 | 7 |
| 238 | 正直 | 1 | 2 | 3 | 4 | 5 | 6 | 7 |
| 239 | 廉洁 | 1 | 2 | 3 | 4 | 5 | 6 | 7 |
| 240 | 清正 | 1 | 2 | 3 | 4 | 5 | 6 | 7 |
| 241 | 热心 | 1 | 2 | 3 | 4 | 5 | 6 | 7 |
| 242 | 实在 | 1 | 2 | 3 | 4 | 5 | 6 | 7 |
| 243 | 骄傲 | 1 | 2 | 3 | 4 | 5 | 6 | 7 |
| 244 | 粗心 | 1 | 2 | 3 | 4 | 5 | 6 | 7 |
| 245 | 敷衍 | 1 | 2 | 3 | 4 | 5 | 6 | 7 |
| 246 | 小气 | 1 | 2 | 3 | 4 | 5 | 6 | 7 |
| 247 | 麻木 | 1 | 2 | 3 | 4 | 5 | 6 | 7 |
| 248 | 肮脏 | 1 | 2 | 3 | 4 | 5 | 6 | 7 |
