# Supplementary material for: The influence of gender stereotypes on gender judgement and impression evaluation based on face and voice
Source: PeerJ. 2025 Jan 31;13:e18900. doi: 10.7717/peerj.18900 (PMC11789659; doi:10.7717/peerj.18900)
Supplement: Supplemental Information 11 [file peerj-13-18900-s011.docx]

**Gender Trait Stereotypy Questionnaire**

Sex___________ Age___________ Grade___________ Major_____________

Dear Student,

Below is a list of words that are often used to describe characteristics associated with males or females. Please rate the extent to which you believe these words are suitable for describing males or females. On this scale, 1 represents the strongest degree of masculinity, that is "very masculine"; 2 indicates "somewhat masculine"; and so on, with 3 indicating a lesser degree of masculinity; 4 signifies "neutral," meaning the trait is applicable to both genders; 5 suggests a leaning towards femininity; 6 indicates "somewhat feminine"; and 7 represents the strongest degree of femininity, that is "very feminine". There is no right or wrong answer, as this is for research purposes only. Please rate each term according to your genuine opinion and mark the corresponding score with a "√".

| Number Very Masculine Neutral Very Feminine | | | | | | | | |
| --- | --- | --- | --- | --- | --- | --- | --- | --- |
| 1 | Tall | 1 | 2 | 3 | 4 | 5 | 6 | 7 |
| 2 | Loving | 1 | 2 | 3 | 4 | 5 | 6 | 7 |
| 3 | Quiet | 1 | 2 | 3 | 4 | 5 | 6 | 7 |
| 4 | Conservative | 1 | 2 | 3 | 4 | 5 | 6 | 7 |
| 5 | Negative | 1 | 2 | 3 | 4 | 5 | 6 | 7 |
| 6 | Irritable | 1 | 2 | 3 | 4 | 5 | 6 | 7 |
| 7 | Passive | 1 | 2 | 3 | 4 | 5 | 6 | 7 |
| 8 | Silent | 1 | 2 | 3 | 4 | 5 | 6 | 7 |
| 9 | Impulsive | 1 | 2 | 3 | 4 | 5 | 6 | 7 |
| 10 | Rough | 1 | 2 | 3 | 4 | 5 | 6 | 7 |
| 11 | Generous | 1 | 2 | 3 | 4 | 5 | 6 | 7 |
| 12 | Humble | 1 | 2 | 3 | 4 | 5 | 6 | 7 |
| 13 | Independence | 1 | 2 | 3 | 4 | 5 | 6 | 7 |
| 14 | Emotional | 1 | 2 | 3 | 4 | 5 | 6 | 7 |
| 15 | Strong | 1 | 2 | 3 | 4 | 5 | 6 | 7 |
| 16 | ‌Unswerving | 1 | 2 | 3 | 4 | 5 | 6 | 7 |
| 17 | Uprighteous | 1 | 2 | 3 | 4 | 5 | 6 | 7 |
| 18 | Philanthropic | 1 | 2 | 3 | 4 | 5 | 6 | 7 |
| 19 | Attack | 1 | 2 | 3 | 4 | 5 | 6 | 7 |
| 20 | Caring | 1 | 2 | 3 | 4 | 5 | 6 | 7 |
| 21 | Bonafide | 1 | 2 | 3 | 4 | 5 | 6 | 7 |
| 22 | Unwavering | 1 | 2 | 3 | 4 | 5 | 6 | 7 |
| 23 | Overrule | 1 | 2 | 3 | 4 | 5 | 6 | 7 |
| 24 | Domineering | 1 | 2 | 3 | 4 | 5 | 6 | 7 |
| 25 | Composed | 1 | 2 | 3 | 4 | 5 | 6 | 7 |
| 26 | Mature | 1 | 2 | 3 | 4 | 5 | 6 | 7 |
| 27 | High | 1 | 2 | 3 | 4 | 5 | 6 | 7 |
| 28 | Daring | 1 | 2 | 3 | 4 | 5 | 6 | 7 |
| 29 | Virtuous | 1 | 2 | 3 | 4 | 5 | 6 | 7 |
| 30 | Filial | 1 | 2 | 3 | 4 | 5 | 6 | 7 |
| 31 | Shy | 1 | 2 | 3 | 4 | 5 | 6 | 7 |
| 32 | Embarrassed | 1 | 2 | 3 | 4 | 5 | 6 | 7 |
| 33 | Gorgeous | 1 | 2 | 3 | 4 | 5 | 6 | 7 |
| 34 | Refined | 1 | 2 | 3 | 4 | 5 | 6 | 7 |
| 35 | Exquisite | 1 | 2 | 3 | 4 | 5 | 6 | 7 |
| 36 | Cultured | 1 | 2 | 3 | 4 | 5 | 6 | 7 |
| 37 | Dependent | 1 | 2 | 3 | 4 | 5 | 6 | 7 |
| 38 | Relying | 1 | 2 | 3 | 4 | 5 | 6 | 7 |
| 39 | Gentle | 1 | 2 | 3 | 4 | 5 | 6 | 7 |
| 40 | Lowly | 1 | 2 | 3 | 4 | 5 | 6 | 7 |
| 41 | Contentious | 1 | 2 | 3 | 4 | 5 | 6 | 7 |
| 42 | Savage | 1 | 2 | 3 | 4 | 5 | 6 | 7 |
| 43 | Adventurous | 1 | 2 | 3 | 4 | 5 | 6 | 7 |
| 44 | Hostile | 1 | 2 | 3 | 4 | 5 | 6 | 7 |
| 45 | Unrefined | 1 | 2 | 3 | 4 | 5 | 6 | 7 |
| 46 | Challenging | 1 | 2 | 3 | 4 | 5 | 6 | 7 |
| 47 | Fiendish | 1 | 2 | 3 | 4 | 5 | 6 | 7 |
| 48 | Fury | 1 | 2 | 3 | 4 | 5 | 6 | 7 |
| 49 | Cautious | 1 | 2 | 3 | 4 | 5 | 6 | 7 |
| 50 | Enduring | 1 | 2 | 3 | 4 | 5 | 6 | 7 |
| 51 | Escapable | 1 | 2 | 3 | 4 | 5 | 6 | 7 |
| 52 | Affectionate | 1 | 2 | 3 | 4 | 5 | 6 | 7 |
| 53 | Tolerant | 1 | 2 | 3 | 4 | 5 | 6 | 7 |
| 54 | Straightforward‌ | 1 | 2 | 3 | 4 | 5 | 6 | 7 |
| 55 | Friendly | 1 | 2 | 3 | 4 | 5 | 6 | 7 |
| 56 | Humor | 1 | 2 | 3 | 4 | 5 | 6 | 7 |
| 57 | Artless | 1 | 2 | 3 | 4 | 5 | 6 | 7 |
| 58 | Unbridled | 1 | 2 | 3 | 4 | 5 | 6 | 7 |
| 59 | Fearless | 1 | 2 | 3 | 4 | 5 | 6 | 7 |
| 60 | Valiant | 1 | 2 | 3 | 4 | 5 | 6 | 7 |
| 61 | Proactive | 1 | 2 | 3 | 4 | 5 | 6 | 7 |
| 62 | Radical | 1 | 2 | 3 | 4 | 5 | 6 | 7 |
| 63 | Short-tempered | 1 | 2 | 3 | 4 | 5 | 6 | 7 |
| 64 | Resolute | 1 | 2 | 3 | 4 | 5 | 6 | 7 |
| 65 | Strong | 1 | 2 | 3 | 4 | 5 | 6 | 7 |
| 66 | Resilient | 1 | 2 | 3 | 4 | 5 | 6 | 7 |
| 67 | Robust | 1 | 2 | 3 | 4 | 5 | 6 | 7 |
| 68 | Ambitious | 1 | 2 | 3 | 4 | 5 | 6 | 7 |
| 69 | Competitive | 1 | 2 | 3 | 4 | 5 | 6 | 7 |
| 70 | Open | 1 | 2 | 3 | 4 | 5 | 6 | 7 |
| 71 | Broad-minded | 1 | 2 | 3 | 4 | 5 | 6 | 7 |
| 72 | Objective | 1 | 2 | 3 | 4 | 5 | 6 | 7 |
| 73 | Stout | 1 | 2 | 3 | 4 | 5 | 6 | 7 |
| 74 | Calm | 1 | 2 | 3 | 4 | 5 | 6 | 7 |
| 75 | Reasonable | 1 | 2 | 3 | 4 | 5 | 6 | 7 |
| 76 | Unassuming | 1 | 2 | 3 | 4 | 5 | 6 | 7 |
| 77 | Powerful | 1 | 2 | 3 | 4 | 5 | 6 | 7 |
| 78 | Sturdy | 1 | 2 | 3 | 4 | 5 | 6 | 7 |
| 79 | Dominant | 1 | 2 | 3 | 4 | 5 | 6 | 7 |
| 80 | Muscular | 1 | 2 | 3 | 4 | 5 | 6 | 7 |
| 81 | Sovereign | 1 | 2 | 3 | 4 | 5 | 6 | 7 |
| 82 | Authoritative | 1 | 2 | 3 | 4 | 5 | 6 | 7 |
| 83 | Educated | 1 | 2 | 3 | 4 | 5 | 6 | 7 |
| 84 | Unrestrained | 1 | 2 | 3 | 4 | 5 | 6 | 7 |
| 85 | Taciturn | 1 | 2 | 3 | 4 | 5 | 6 | 7 |
| 86 | Sagacious | 1 | 2 | 3 | 4 | 5 | 6 | 7 |
| 87 | Smart | 1 | 2 | 3 | 4 | 5 | 6 | 7 |
| 88 | Tenacious | 1 | 2 | 3 | 4 | 5 | 6 | 7 |
| 89 | Stark | 1 | 2 | 3 | 4 | 5 | 6 | 7 |
| 90 | Majestic | 1 | 2 | 3 | 4 | 5 | 6 | 7 |
| 91 | Great | 1 | 2 | 3 | 4 | 5 | 6 | 7 |
| 92 | Steady | 1 | 2 | 3 | 4 | 5 | 6 | 7 |
| 93 | Carefree | 1 | 2 | 3 | 4 | 5 | 6 | 7 |
| 94 | Dashing | 1 | 2 | 3 | 4 | 5 | 6 | 7 |
| 95 | Ferocious | 1 | 2 | 3 | 4 | 5 | 6 | 7 |
| 96 | Stern | 1 | 2 | 3 | 4 | 5 | 6 | 7 |
| 97 | Masculine | 1 | 2 | 3 | 4 | 5 | 6 | 7 |
| 98 | Cheerful | 1 | 2 | 3 | 4 | 5 | 6 | 7 |
| 99 | Handsome | 1 | 2 | 3 | 4 | 5 | 6 | 7 |
| 100 | Heroic | 1 | 2 | 3 | 4 | 5 | 6 | 7 |
| 101 | Resolute | 1 | 2 | 3 | 4 | 5 | 6 | 7 |
| 102 | Courageous | 1 | 2 | 3 | 4 | 5 | 6 | 7 |
| 103 | Assertive | 1 | 2 | 3 | 4 | 5 | 6 | 7 |
| 104 | Decisive | 1 | 2 | 3 | 4 | 5 | 6 | 7 |
| 105 | Happy | 1 | 2 | 3 | 4 | 5 | 6 | 7 |
| 106 | Erudite | 1 | 2 | 3 | 4 | 5 | 6 | 7 |
| 107 | Rational | 1 | 2 | 3 | 4 | 5 | 6 | 7 |
| 108 | Sincere | 1 | 2 | 3 | 4 | 5 | 6 | 7 |
| 109 | Self-respecting | 1 | 2 | 3 | 4 | 5 | 6 | 7 |
| 110 | Talented | 1 | 2 | 3 | 4 | 5 | 6 | 7 |
| 111 | Frank | 1 | 2 | 3 | 4 | 5 | 6 | 7 |
| 112 | Studious | 1 | 2 | 3 | 4 | 5 | 6 | 7 |
| 113 | Diligent | 1 | 2 | 3 | 4 | 5 | 6 | 7 |
| 114 | Polite | 1 | 2 | 3 | 4 | 5 | 6 | 7 |
| 115 | Generous | 1 | 2 | 3 | 4 | 5 | 6 | 7 |
| 116 | Conscientious | 1 | 2 | 3 | 4 | 5 | 6 | 7 |
| 117 | Enthusiastic | 1 | 2 | 3 | 4 | 5 | 6 | 7 |
| 118 | Hardworking | 1 | 2 | 3 | 4 | 5 | 6 | 7 |
| 119 | Optimistic | 1 | 2 | 3 | 4 | 5 | 6 | 7 |
| 120 | Dedicated | 1 | 2 | 3 | 4 | 5 | 6 | 7 |
| 121 | Modest | 1 | 2 | 3 | 4 | 5 | 6 | 7 |
| 122 | Leniency | 1 | 2 | 3 | 4 | 5 | 6 | 7 |
| 123 | Self-esteeming | 1 | 2 | 3 | 4 | 5 | 6 | 7 |
| 124 | Self-love | 1 | 2 | 3 | 4 | 5 | 6 | 7 |
| 125 | Independent | 1 | 2 | 3 | 4 | 5 | 6 | 7 |
| 126 | Solid | 1 | 2 | 3 | 4 | 5 | 6 | 7 |
| 127 | Serene | 1 | 2 | 3 | 4 | 5 | 6 | 7 |
| 128 | Charitable | 1 | 2 | 3 | 4 | 5 | 6 | 7 |
| 129 | Beautiful | 1 | 2 | 3 | 4 | 5 | 6 | 7 |
| 130 | Auspicious | 1 | 2 | 3 | 4 | 5 | 6 | 7 |
| 131 | Candid | 1 | 2 | 3 | 4 | 5 | 6 | 7 |
| 132 | Malicious | 1 | 2 | 3 | 4 | 5 | 6 | 7 |
| 133 | Flattering | 1 | 2 | 3 | 4 | 5 | 6 | 7 |
| 134 | Despicable | 1 | 2 | 3 | 4 | 5 | 6 | 7 |
| 135 | Contemptible | 1 | 2 | 3 | 4 | 5 | 6 | 7 |
| 136 | Sad | 1 | 2 | 3 | 4 | 5 | 6 | 7 |
| 137 | Angry | 1 | 2 | 3 | 4 | 5 | 6 | 7 |
| 138 | Sarcastic | 1 | 2 | 3 | 4 | 5 | 6 | 7 |
| 139 | Ungrateful | 1 | 2 | 3 | 4 | 5 | 6 | 7 |
| 140 | Isolated | 1 | 2 | 3 | 4 | 5 | 6 | 7 |
| 141 | Vicious | 1 | 2 | 3 | 4 | 5 | 6 | 7 |
| 142 | Muddled | 1 | 2 | 3 | 4 | 5 | 6 | 7 |
| 143 | Treacherous | 1 | 2 | 3 | 4 | 5 | 6 | 7 |
| 144 | Mean | 1 | 2 | 3 | 4 | 5 | 6 | 7 |
| 145 | Impetuous | 1 | 2 | 3 | 4 | 5 | 6 | 7 |
| 146 | Upset | 1 | 2 | 3 | 4 | 5 | 6 | 7 |
| 147 | Submissive | 1 | 2 | 3 | 4 | 5 | 6 | 7 |
| 148 | Deceitful | 1 | 2 | 3 | 4 | 5 | 6 | 7 |
| 149 | Failure | 1 | 2 | 3 | 4 | 5 | 6 | 7 |
| 150 | Lost | 1 | 2 | 3 | 4 | 5 | 6 | 7 |
| 151 | Disappointed | 1 | 2 | 3 | 4 | 5 | 6 | 7 |
| 152 | Agony | 1 | 2 | 3 | 4 | 5 | 6 | 7 |
| 153 | Arrogant | 1 | 2 | 3 | 4 | 5 | 6 | 7 |
| 154 | Confident | 1 | 2 | 3 | 4 | 5 | 6 | 7 |
| 155 | Charming | 1 | 2 | 3 | 4 | 5 | 6 | 7 |
| 156 | Radiant | 1 | 2 | 3 | 4 | 5 | 6 | 7 |
| 157 | Innocent | 1 | 2 | 3 | 4 | 5 | 6 | 7 |
| 158 | Elegant | 1 | 2 | 3 | 4 | 5 | 6 | 7 |
| 159 | Dignified | 1 | 2 | 3 | 4 | 5 | 6 | 7 |
| 160 | Plump | 1 | 2 | 3 | 4 | 5 | 6 | 7 |
| 161 | Noble | 1 | 2 | 3 | 4 | 5 | 6 | 7 |
| 162 | Coy | 1 | 2 | 3 | 4 | 5 | 6 | 7 |
| 163 | Bashful | 1 | 2 | 3 | 4 | 5 | 6 | 7 |
| 164 | Amiable | 1 | 2 | 3 | 4 | 5 | 6 | 7 |
| 165 | Beauty | 1 | 2 | 3 | 4 | 5 | 6 | 7 |
| 166 | Dainty | 1 | 2 | 3 | 4 | 5 | 6 | 7 |
| 167 | Stoic | 1 | 2 | 3 | 4 | 5 | 6 | 7 |
| 168 | Cute | 1 | 2 | 3 | 4 | 5 | 6 | 7 |
| 169 | Stunning | 1 | 2 | 3 | 4 | 5 | 6 | 7 |
| 170 | Restrained | 1 | 2 | 3 | 4 | 5 | 6 | 7 |
| 171 | Agile | 1 | 2 | 3 | 4 | 5 | 6 | 7 |
| 172 | Fragrant | 1 | 2 | 3 | 4 | 5 | 6 | 7 |
| 173 | Timid | 1 | 2 | 3 | 4 | 5 | 6 | 7 |
| 174 | Patient | 1 | 2 | 3 | 4 | 5 | 6 | 7 |
| 175 | Good-looking | 1 | 2 | 3 | 4 | 5 | 6 | 7 |
| 176 | Graceful | 1 | 2 | 3 | 4 | 5 | 6 | 7 |
| 177 | Spicy | 1 | 2 | 3 | 4 | 5 | 6 | 7 |
| 178 | Reserved | 1 | 2 | 3 | 4 | 5 | 6 | 7 |
| 179 | Alluring | 1 | 2 | 3 | 4 | 5 | 6 | 7 |
| 180 | Pretty | 1 | 2 | 3 | 4 | 5 | 6 | 7 |
| 181 | Light | 1 | 2 | 3 | 4 | 5 | 6 | 7 |
| 182 | Soft | 1 | 2 | 3 | 4 | 5 | 6 | 7 |
| 183 | Playful | 1 | 2 | 3 | 4 | 5 | 6 | 7 |
| 184 | Softness | 1 | 2 | 3 | 4 | 5 | 6 | 7 |
| 185 | Weak | 1 | 2 | 3 | 4 | 5 | 6 | 7 |
| 186 | Kind | 1 | 2 | 3 | 4 | 5 | 6 | 7 |
| 187 | Submissive | 1 | 2 | 3 | 4 | 5 | 6 | 7 |
| 188 | Obedient | 1 | 2 | 3 | 4 | 5 | 6 | 7 |
| 189 | Easy-going | 1 | 2 | 3 | 4 | 5 | 6 | 7 |
| 190 | Considerate | 1 | 2 | 3 | 4 | 5 | 6 | 7 |
| 191 | Docile | 1 | 2 | 3 | 4 | 5 | 6 | 7 |
| 192 | Tender | 1 | 2 | 3 | 4 | 5 | 6 | 7 |
| 193 | Meek | 1 | 2 | 3 | 4 | 5 | 6 | 7 |
| 194 | Urbane | 1 | 2 | 3 | 4 | 5 | 6 | 7 |
| 195 | Sophisticated | 1 | 2 | 3 | 4 | 5 | 6 | 7 |
| 196 | ‌Civil | 1 | 2 | 3 | 4 | 5 | 6 | 7 |
| 197 | Delicate | 1 | 2 | 3 | 4 | 5 | 6 | 7 |
| 198 | Attentive | 1 | 2 | 3 | 4 | 5 | 6 | 7 |
| 199 | Enchanting | 1 | 2 | 3 | 4 | 5 | 6 | 7 |
| 200 | Virtuous | 1 | 2 | 3 | 4 | 5 | 6 | 7 |
| 201 | Sheepish‌ | 1 | 2 | 3 | 4 | 5 | 6 | 7 |
| 202 | Dirty | 1 | 2 | 3 | 4 | 5 | 6 | 7 |
| 203 | Shameless | 1 | 2 | 3 | 4 | 5 | 6 | 7 |
| 204 | Narrow-minded | 1 | 2 | 3 | 4 | 5 | 6 | 7 |
| 205 | Vile | 1 | 2 | 3 | 4 | 5 | 6 | 7 |
| 206 | Vulgar | 1 | 2 | 3 | 4 | 5 | 6 | 7 |
| 207 | Brutal | 1 | 2 | 3 | 4 | 5 | 6 | 7 |
| 208 | Inactive | 1 | 2 | 3 | 4 | 5 | 6 | 7 |
| 209 | Hypocritical | 1 | 2 | 3 | 4 | 5 | 6 | 7 |
| 210 | Treacherous | 1 | 2 | 3 | 4 | 5 | 6 | 7 |
| 211 | Ignorant | 1 | 2 | 3 | 4 | 5 | 6 | 7 |
| 212 | Self-important | 1 | 2 | 3 | 4 | 5 | 6 | 7 |
| 213 | Complacent | 1 | 2 | 3 | 4 | 5 | 6 | 7 |
| 214 | Selfish | 1 | 2 | 3 | 4 | 5 | 6 | 7 |
| 215 | Stupid | 1 | 2 | 3 | 4 | 5 | 6 | 7 |
| 216 | Self-centered | 1 | 2 | 3 | 4 | 5 | 6 | 7 |
| 217 | Slovenly | 1 | 2 | 3 | 4 | 5 | 6 | 7 |
| 218 | Smudged | 1 | 2 | 3 | 4 | 5 | 6 | 7 |
| 219 | Terrible | 1 | 2 | 3 | 4 | 5 | 6 | 7 |
| 220 | Sarcastic | 1 | 2 | 3 | 4 | 5 | 6 | 7 |
| 221 | Mocking | 1 | 2 | 3 | 4 | 5 | 6 | 7 |
| 222 | Disdainful | 1 | 2 | 3 | 4 | 5 | 6 | 7 |
| 223 | Contemptuous | 1 | 2 | 3 | 4 | 5 | 6 | 7 |
| 224 | Bitter | 1 | 2 | 3 | 4 | 5 | 6 | 7 |
| 225 | Mischievous | 1 | 2 | 3 | 4 | 5 | 6 | 7 |
| 226 | Despair | 1 | 2 | 3 | 4 | 5 | 6 | 7 |
| 227 | Responsible | 1 | 2 | 3 | 4 | 5 | 6 | 7 |
| 228 | Strict | 1 | 2 | 3 | 4 | 5 | 6 | 7 |
| 229 | Holy | 1 | 2 | 3 | 4 | 5 | 6 | 7 |
| 230 | Selfless | 1 | 2 | 3 | 4 | 5 | 6 | 7 |
| 231 | Compassionate | 1 | 2 | 3 | 4 | 5 | 6 | 7 |
| 232 | Assiduous | 1 | 2 | 3 | 4 | 5 | 6 | 7 |
| 233 | Capable | 1 | 2 | 3 | 4 | 5 | 6 | 7 |
| 234 | Magnanimous | 1 | 2 | 3 | 4 | 5 | 6 | 7 |
| 235 | Kind-hearted | 1 | 2 | 3 | 4 | 5 | 6 | 7 |
| 236 | Mild | 1 | 2 | 3 | 4 | 5 | 6 | 7 |
| 237 | Moral | 1 | 2 | 3 | 4 | 5 | 6 | 7 |
| 238 | Upright | 1 | 2 | 3 | 4 | 5 | 6 | 7 |
| 239 | Honest | 1 | 2 | 3 | 4 | 5 | 6 | 7 |
| 240 | Incorruptible | 1 | 2 | 3 | 4 | 5 | 6 | 7 |
| 241 | Zealous | 1 | 2 | 3 | 4 | 5 | 6 | 7 |
| 242 | Practical | 1 | 2 | 3 | 4 | 5 | 6 | 7 |
| 243 | Proud | 1 | 2 | 3 | 4 | 5 | 6 | 7 |
| 244 | Careless | 1 | 2 | 3 | 4 | 5 | 6 | 7 |
| 245 | Perfunctory | 1 | 2 | 3 | 4 | 5 | 6 | 7 |
| 246 | Stingy | 1 | 2 | 3 | 4 | 5 | 6 | 7 |
| 247 | Insensitive | 1 | 2 | 3 | 4 | 5 | 6 | 7 |
| 248 | Mess | 1 | 2 | 3 | 4 | 5 | 6 | 7 |
